# Influence of the Mesoporosity of Hierarchical ZSM-5 in Toluene Alkylation by Methanol

**DOI:** 10.3390/ma16216872

**Published:** 2023-10-26

**Authors:** Lucie Desmurs, Claudia Cammarano, Olinda Gimello, Anne Galarneau, Vasile Hulea

**Affiliations:** ICGM, University of Montpellier, CNRS, ENSCM, 34293 Montpellier, France; lucie.desmurs@enscm.fr (L.D.); claudia.cammarano@enscm.fr (C.C.); olinda.gimelo@enscm.fr (O.G.); anne.galarneau@enscm.fr (A.G.)

**Keywords:** hierarchical ZSM-5, alkylation, toluene, methanol, para-xylene

## Abstract

Among the different strategies to design highly shape-selective ZSM-5 to obtain para-xylene through toluene alkylation with methanol, the introduction of mesopores to increase reactant and product diffusion has been proposed but barely studied. In this study, we prepared mesoporous ZSM-5 catalysts, named ZSM5-MT(x), from commercial ZSM-5 (Si/Al = 15), using a two-step micelle-templating procedure with octadecyltrimethylammonium bromide as a surfactant in basic medium (x = NaOH/Si). These materials were used as catalysts for the alkylation of toluene by methanol at a low contact time to avoid thermodynamic equilibrium of the xylene isomers. Compared to the parent ZSM-5, the mesoporous ZSM5-MT(x) catalysts did not improve the para-xylene selectivity, revealing that the strategy of increasing diffusion in the catalyst is not a good strategy to follow. However, ZSM5-MT(0.5) showed less deactivation on stream than the parent ZSM-5. Therefore, introducing mesopores to ZSM-5 could be interesting to explore, combined with another strategy of shape selectivity, such as the passivation of the external surface acidity.

## 1. Introduction

Zeolites are well-known heterogeneous catalysts that have an indispensable role in the chemical industry [1,2,3]. Their specific microporous system confers them with unique acidic properties and shape selectivity. By tuning their porous architectures, framework compositions, and crystal morphologies, zeolites exhibit unprecedentedly high performances in many processes [3]. A relevant example is toluene alkylation with methanol to produce para-xylene, a major intermediate used to produce high-valued polymers. Medium pore zeolites, such as ZSM-5 (MFI structure, 10-ring channel system), are the most suitable catalysts for toluene alkylation [4]. However, the shape-selective capability of ZSM-5 to produce para-xylene from toluene alkylation continues to be one of the industrial challenges. ZSM-5 features two types of interconnected channels: a straight channel of an ellipsoidal cross-section (0.52 × 0.56 nm) and sinusoidal channels of an almost circular cross-section (0.54 × 0.56 nm). The intersection of the channels gives rise to large cavities of ~0.8 nm.

Because of its linear shape, para-xylene diffuses in ZSM-5 channels more rapidly than ortho- and meta-xylene. For instance, the experimental relative diffusion coefficients of para-/meta-/ortho-xylene were 123/1/16 [5].

There are different advised strategies to design highly shape-selective ZSM-5 to obtain para-xylene through toluene alkylation with methanol [6]:

(1) Passivation of the external acid sites by coking [7] or with modifiers as TEOS or P [8,9,10] in order to avoid the secondary para-xylene isomerization to ortho- and meta-xylene on the external surface.
(2) Fine tuning of the pore size of ZSM-5 to improve its shape selective capability in a primary reaction. This has been already proved with the synthesis of ZSM-5 crystals by maximizing the pore opening through sinusoidal channels [11].
(3) Better control of the Al distribution in the framework of ZSM-5. For example, a low Al concentration at the surface favors the para-xylene production [11].
(4) Increase in the crystal size (10 micron) to lengthen the pathway of meta-xylene and ortho-xylene and favor their isomerization into para-xylene [12,13].
(5) Reduction of the contact time to limit the isomerization of para-xylene [14].
(6) Introduction of mesopores to increase reactant and product diffusion [15].

Among these strategies, the influence of mesopores in ZSM-5 crystals towards para-xylene selectivity has barely been evaluated in the alkylation of toluene. Lyu et al. [16] synthesized a hierarchical ZSM-5 (Si/Al = 180) characterized by particles of 200–300 nm formed by an aggregation of round crystals of 10–50 nm and mesopores with pore diameters from 5 to 30 nm and carried out the alkylation of benzene with methanol (benzene/methanol = 1/1) at 400 °C. The hierarchical ZSM-5 showed good stability on stream over time, but the selectivity in para-xylene remained close to the thermodynamical equilibrium. Li et al. [17] compared the ZSM-5 nanosheets (Si/Al = 22) performances with those of ZSM-5 nanocrystals (500 nm particle size formed by an aggregation of 50 nm nanocrystals), alkali treated ZSM-5 (AT) and ZSM-5 microcrystals (2–4 μm). ZSM-5 (AT) featured mesopores, with a mesopore volume of 0.5 mL/g. The toluene alkylation was carried out at 420 °C with a toluene/methanol ratio of 6/1 and with a large excess of H_2_ and H_2_O. For all the kinds of studied ZSM-5 (with or without mesopores) the para-xylene selectivity did not exceed the thermodynamic equilibrium (24%).

Recently, we synthesized a family of mesoporous ZSM-5 catalysts with well-defined mesopores (narrow pore size distribution of 4 nm diameter) by micelle-templating starting from a commercial ZSM-5 (Si/Al = 15) featuring nanocrystals (50–100 nm) [18]. By increasing the NaOH/Si ratio in the synthesis, different features of mesoporous ZSM-5 were noticed: (i) the micropore volume remained constant until NaOH/Si = 0.3 and then decreased, while the mesopore volume increased continuously [18], (ii) the number Brønsted acid decreased [19], (iii) the strength of the acid sites remained constant until NaOH/Si = 0.38 and then decreased [19]. Two catalytic test reactions (alkylation of phenol by *tert*-butyl alcohol and esterification of benzyl alcohol with hexanoic acid) were run and demonstrated that the best compromise between large molecule accessibility and strong Brønsted acidity was reached for mesoporous ZSM-5 catalysts synthesized with 0.38 ≤ NaOH/Si ≤ 0.5 [19].

In the present study, we have examined the catalytic conditions of toluene alkylation by methanol to be out of thermodynamic equilibrium and favor the para-xylene formation with the parent ZSM-5. In order to examine the effect of increasing diffusivity, the entire family of mesoporous ZSM-5 with increasing mesopore volumes was used. The aim of this work is to verify if the strategy of introducing mesopores in ZSM-5 is appropriate for increasing the catalytic behavior of these type of catalysts in toluene reactions with methanol. The selectivity in para-xylene and the catalyst lifetime exhibited by the mesoporous zeolites were compared to those sowed by the parent ZSM-5.

## 2. Experimental Section

### 2.1. Synthesis of Mesoporous ZSM-5

Mesoporous ZSM-5 were prepared via a 2-step micelle-templating procedure adapted from Goto et al. [20], starting with a commercial ZSM-5 (Si/Al = 15). In a typical synthesis, 2.5 g of NH_4_-ZSM-5 (CBV3024E Zeolyst, crystal size of about 100 nm) were treated with sodium hydroxide (Sigma-Aldrich, Saint-Quentin-Fallavier, France) and octadecyltrimethylammonium bromide (C_18_TAB, Sigma-Aldrich), with the following ratios, assuming zeolite in the calculation as pure silica: 1 SiO_2_: 0.1 C_18_TAB: x NaOH: 56 H_2_O with 0.30 ≤ x ≤ 0.70. First, the aqueous solution of NaOH and C_18_TAB was prepared in a water bath at 50 °C to optimize surfactant dissolution, and then the zeolite powder was added under stirring. After dispersion for 20 min, the mixture was poured into a Teflon lined stainless steel autoclave for a 24 h first step at 110 °C, followed by pH adjustment to pH 8.5 with 2.0 M HCl solution and a second 24 h step at 110 °C. The solid was recovered by filtration, washed with deionized water until neutral pH, dried overnight at 100 °C, and calcined 2 h at 350 °C and 8 h at 550 °C (0.5 °C min^−1^) to remove the surfactant. The synthetized materials were designated ZSM5-MT(x), with x equal to the NaOH/Si synthesis ratio. The acidic form of catalysts was obtained by ionic exchange with an aqueous 0.1 M NH_4_NO_3_ solution (100 mL per g solid) at 90 °C under reflux for 1 h. The procedure was repeated 3 times with filtration in between, and then the material was washed with water (100 mL per g solid), dried at 80 °C, and calcined at 550 °C for 8 h to liberate ammonia and obtain the H^+^ form.

### 2.2. Material Characterization

The textural properties were analyzed by N_2_ sorption at 77 K, and 100 mg of materials were outgassed under vacuum at 523 K for 6 h prior to nitrogen sorption analysis at 77 K using a Micromeritics Tristar (made by Micromeritics Instrument Corporation, Norcross, GA, USA) apparatus. The crystalline phase and mesoporous order were followed by X-ray diffraction (XRD) on a D8 Bruker device (Cu Kα radiation) in the ranges of 2θ = 4–50° and 0.5–6°, respectively, with a 0.2° angular step. Transmission electron microscopy (TEM) images were recorded using a JEOL 1200 EX2 (JEOL Ltd., Tokyo, Japan) microscope operating at 100 kV at “Plateau Technique du Pole Chimie Balard Montpellier”. Determination of the Brønsted and Lewis acidity was performed by FTIR/pyridine on a Nicolet 5700 instrument (Thermo Fisher Scientific Co, Waltham, MA, USA) [19]. The material was first compressed (0.5 T) to form a self-supported pellet (2 cm^−2^, 10–30 mg) and heated at 823 K under air flow (60 mL min^−1^). The sample was then outgassed (10^−5^ bar) at 473 K for 1 h prior to registering the first FTIR spectrum. The sample was cooled at 423 K and exposed to pyridine (1.5 mbar) for 5 min and outgassed (10^−5^ bar) to remove physisorbed pyridine. The quantification of the Brønsted and Lewis acid sites was carried out by integration of the bands at 1545 and 1455 cm^−1^, respectively, with the following extinction coefficients: ε_1545_ = 1.13 and ε_1455_ = 1.28 cm mol^−1^. The acidity strength was probed with NH_3_-TPD on a Micromeritics Autochem II with 50 mg of samples. The particle size of the powders was controlled by compressing (2 T) 200 mg of powder into pellets with a 16 mm diameter for 1 min with a SPECAC 15 t press (Thermo Fisher Scientific Co, Waltham, MA, USA) and then grinding and sieving the pellets between 150 and 250 µm. A 2 h calcination step at 550 °C allowed for the sample surface preparation before ammonia adsorption at 373K for 40 min with 5% NH_3_ in He. Desorption was carried out with a 25 cm^3^/min He flow at 10 °C/min to 600 °C. Temperature ramping started when the TCD signal was stable and desorption of weakly physisorbed ammonia was over. A calibration of the TCD signal allowed for the data treatment and calculation of acid site concentration. The Si/Al molar ratio of the ZSM5-MT(x) was calculated by ICP-OES analysis.

### 2.3. Alkylation of Toluene by Methanol

Toluene alkylation was carried out in a continuous gas flow fixed-bed reactor. The gas composition was evaluated by gas chromatography (GC-Agilent 7890A, Agilent Technologies, Inc., Wilmington, NC, USA, equipped with a ZBWAX column of 30 m × 250 µm × 0.25 µm and a FID detector). In a typical test, the reactor was filled with: 10 mg of catalyst (150–250 µm particles size) previously mixed with 140 mg of quartz, and glass beads filling to the top. After a preliminary 8 h calcination step at 550 °C under 40 mL min^−1^ air flow, the temperature was set at 350 °C, with flowing N_2_. Then, 0.020 mL min^−1^ of liquid toluene and methanol in the desired ratio were fed using a HPLC pump (Jasco PU-4180, Jasco Inc. Easton, PA, USA) and mixed with 80 mL min^−1^ N_2_. A GC separation program allowed for the separation (Figure S1) of light hydrocarbons (HCs), methanol (M), benzene (B), toluene (T), p-/m-/o-xylene (X) isomers, and trimethylbenzene (TMB).

The conversion and selectivity were calculated according to the following equations, using the molar quantity of each compound determined from the peak area on the chromatogram.

$$\mathrm{Toluene}\;\mathrm{conversion}:\;C_{T}=\frac{B+X+TMB}{T+B+X+TMB}\times 100\;(\%)$$

$$\mathrm{Methanol}\;\mathrm{conversion}:\;C_{M}=\frac{M_{0}-M}{M_{0}}\times 100$$

$$\mathrm{Xylene}\;\mathrm{isomer}\;\mathrm{selectivity}:\;S_{i˗X}=\frac{i˗X}{\sum i˗X}\times 100\;\left(\%\right)$$

where *i-* = p- or m- or o-

$$\mathrm{Aromatic}\;\mathrm{selectivity}:\;S_{P}=\frac{P}{\sum P}\times 100\;(\%)$$

where *p* = B or X or TMB

$$\mathrm{Methanol}\;\mathrm{consumption}\;\mathrm{in}\;\mathrm{alkylation}:\;M_{alk}=\frac{{\dot{T}}_{in}\times C_{T}\times (S_{X}+{2S}_{TMB}-S_{B})}{{\dot{M}}_{in}\times C_{M}}\left(\%\right)$$

$$p-\mathrm{xylene}\;\mathrm{yield}:\;R_{p-X}=C_{T}\times S_{X}\times S_{p-X}\left(\%\right)$$

$$\mathrm{Toluene}\;\mathrm{turnover}\;\mathrm{frequency}:\;TOF=\frac{{{\dot{T}}_{in}\times C}_{T}\times 60}{{m\times n}_{H_{B}^{+}}}\;(h^{-1})$$

$$\mathrm{Normalized}\;\mathrm{ratio}\;\mathrm{of}\;\mathrm{light}\;\mathrm{hydrocarbons}:\;R_{HC}=\frac{HC}{{HC}_{Référence}}$$

where ${\dot{T}}_{in}$ is the toluene feed (mol/min) [17], $n_{H_{B}^{+}}$ is the number of Brønsted acid sites determined by FTIR-pyridine (mol g^−1^), m is the catalyst mass (g) and $R_{HC}$ is the ratio of the light hydrocarbons peak area of the investigated reaction (HC) by the light hydrocarbons peak area of the reference reaction (HC_reference_).

## 3. Results and Discussion

### 3.1. Alkylation of Toluene by Methanol on ZSM-5 (Si/Al = 15)

**Scheme 1:**
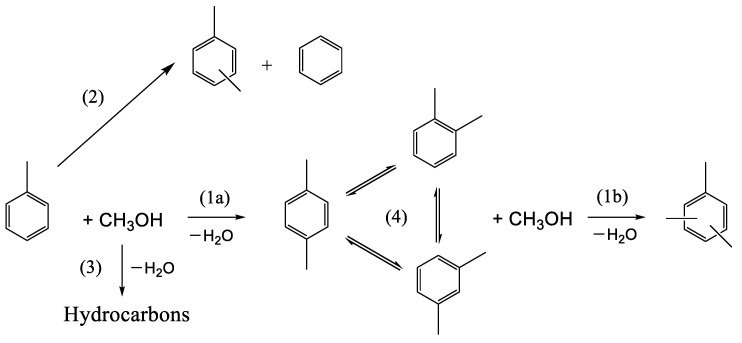
Main reactions involved in the alkylation of toluene by methanol. (1a) Toluene alkylation; (1b) xylene alkylation; (2) toluene disproportionation; (3) methanol to hydrocarbons; (4) xylene isomerization.

The main reaction between toluene and methanol is alkylation, giving rise to xylenes (Scheme 1). This reaction, which is catalyzed by the Brønsted acid sites of the catalyst, is accompanied by several side reactions, such as xylene isomerization, toluene disproportionation, the transformation of methanol into light hydrocarbons, and the poly-methylation of toluene (giving, for example, TMB), etc. (Scheme 1). The reaction conditions must be chosen to maximize the yield of xylenes and avoid the formation of side molecules.

The reaction conditions, i.e., temperature, toluene/methanol ratio, and contact time, were first examined with the commercial ZSM-5 (Si/Al = 15). The alkylation of toluene is effective for temperatures above 300 °C. The conversion of toluene increases with increases in the catalyst weight and temperature (Table S1). With 200 mg ZSM-5 at 450 °C and a toluene/methanol = 1/2, the conversion of toluene and methanol reached 84 and 100%, respectively, with a selectivity of xylenes of 54%, but a low methanol alkylation efficiency (29%). A high amount of methanol favored the formation of TMB (Table S2). Benzene was detected only as traces under our conditions, signifying that toluene disproportionation did not take place. Note that the industrial disproportionation of toluene to produce benzene and xylene is performed at temperatures higher than 450 °C. With a high quantity of catalysts (200 mg), corresponding to a contact time of 0.20 s, the thermodynamic equilibrium of the p-/m-/o-xylenes was reached (p/m/o = 24/52/24%) for a temperature ranging between 350 and 450 °C. With a lower amount of catalyst (80 mg), corresponding to a contact time of 0.09 s, the ratio of the isomers was p/m/o = 35/39/26% at 350 °C.

Decreasing the amount of methanol (toluene/methanol = 4/1) favored the methanol alkylation efficiency (87%), and the toluene conversion was 17%, but the thermodynamic equilibrium was reached, and the ratio of the isomers was p/m/o = 27/52/21% at 350 °C.

The effect of the contact time (Figure S2) was studied at 350 °C with a molar ratio toluene/methanol = 4/1 to maximize the toluene conversion, favor the methanol alkylation efficiency, and avoid successive alkylation. Different contact times (ratio of catalyst volume by the total gas flow rate) were obtained by using different weights of the catalyst (Table S3). The decrease in the contact time decreased toluene conversion but increased the para-selectivity, which was no longer controlled by the thermodynamic equilibrium. For example, with a small amount of catalyst (10 mg), at a short contact time of 0.03 s, the toluene conversion was 12%, the methanol alkylation efficiency was 63%, and among the xylenes, the percentages of p-, m-, and o-xylene were 61, 25, and 14%, respectively. Under these conditions, the isomerization of the primary compounds was practically avoided. This finding indicates that para-xylene is the primary isomer produced in the toluene alkylation of ZSM-5.

**Scheme 2:**
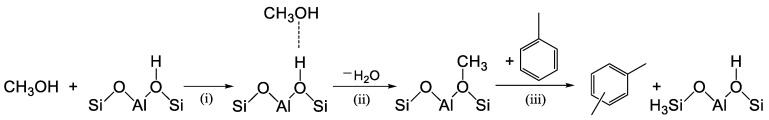
Stepwise mechanisms for methylation of toluene on zeolites with methanol (CH3OH): (i) adsorption of CH3OH on a Brønsted proton; (ii) dehydration of methanol; (iii) toluene methylation.

To support this assertion, we considered the recent theoretical studies (DFT calculations) performed by Chen et al. [21] on ZSM-5 and Parmar et al. [22] on MCM-22 zeolite. For the alkylation of toluene with methanol, they considered a stepwise mechanism, which involves three main steps: (i) adsorption of CH_3_OH on a Brønsted proton; (ii) dehydration of methanol to form surface-bound methoxy species (−OCH_3_), and (iii) methylation, involving the interaction of the methoxy function with toluene (Scheme 2). Methylation was found to be the rate-determining step, with the highest free-energy barrier. The DFT calculation also showed that the methylation reaction of toluene and methanol on ZSM-5 and MCM-22 to form o-, m-, and p-xylene occurs through similar transition states and activation barriers.

For example, for the acid sites placed in the surface pockets of MCM-22 (where there are no spatial constraints), the energy barriers calculated for step (iii) were similar for para, meta, and ortho-xylene (1.15, 1.12, and 1.03 eV, respectively). Thus, p-xylene, o-xylene, and m-xylene are generated with a similar possibility in the primary products.

In contrast, when the transition state selectivity was investigated in the internal sinusoidal channels (0.40 × 0.55 nm) of MCM-22, the barriers for the methyl group attacking a carbon atom on the toluene ring (step (iii)) were 0.88, 1.17, and 1.20 eV for the para, meta, and ortho positions, respectively. This means that in the presence of high spatial constraints, there is an appreciable transition state selectivity for p-xylene formation. Additionally, Chen et al. [21] found that the diffusion barrier for p-xylene is lower than m-xylene in ZSM-5, and the diffusion barrier of m-xylene along the zigzag channel is higher than the barrier of its isomerization to p-xylene, which further promotes the selectivity of p-xylene formation in ZSM-5.

We consider that a similar effect was present in our case, when the toluene alkylation occurred inside the pores of ZSM-5.

### 3.2. Descriptions of Mesoporous ZSM-5 Catalysts

**Figure 1:**
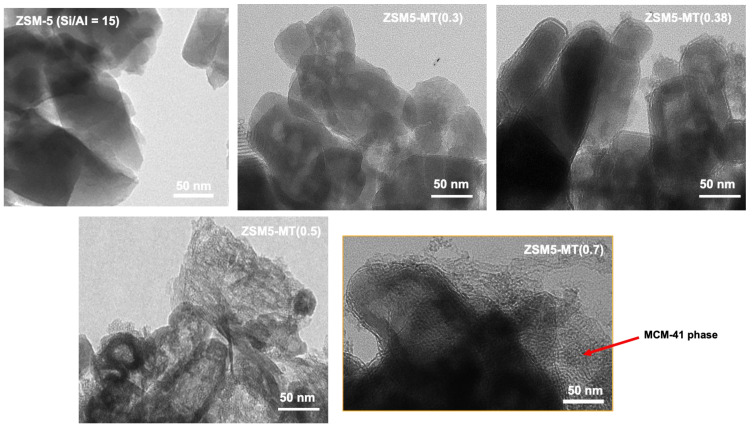
TEM images of parent ZSM-5 and ZSM5-MT(x). The red arrow indicates the presence of an Al-MCM-41- like phase.

Mesoporous ZSM5-MT(x) was synthesized from commercial ZSM-5 (Si/Al = 15) featuring nanocrystals (50–100 nm) by micelle-templating with an increasing amount of x = NaOH/Si in gel synthesis, and characterized by TEM (Figure 1), nitrogen sorption isotherms at 77K (Figure S3), XRD (Figure S4), NH_3_-TPD (Figure S5), and FTIR-Pyridine [19]. The main characteristics (surface areas, pore volumes, acidity) of the mesoporous ZSM-5 catalysts are summarized in Table S4. XRD patterns of the ZSM-5 crystals were observed for all ZSM5-MT(x), with additional diffraction peaks at low angles characteristic of Al-MCM-41-like mesopores ordered with a unit cell parameter of 4.9 nm [19]. TEM pictures show the preservation of the parent nanocrystal morphology for ZSM5-MT(x = 0.3 and 0.38) (Figure 1). For ZSM5-MT(0.5), some Al-MCM-41-like zones were identified, the number of which increased with x to give mostly a Al-MCM-41-like material for ZSM5-MT(0.8). TEM revealed that the ZSM-5 nanocrystals of ZSM5-MT(x) were hollow, and the size of the shells decreased with an increasing x.

The ZSM5-MT(x) characterization (Figure 1) is summarized as follows:

1. ZSM5-MT(0.3) and ZSM5-MT(0.38) feature hollow boxes of ~100 nm of ZSM-5 crystals surrounded by a layer of closely attached Al-MCM-41-like phase.
2. ZSM5-MT(0.5) is composed of hollow boxes of ZSM-5 with some open windows, surrounded by a closely attached layer of Al-MCM-41-like phase; some Al-MCM-41-like phase also extended in between the nanocrystals.
3. ZSM5-MT(0.6) and ZSM5-MT(0.7) are composites of two distinct phases: hollow boxes of ZSM-5 with some open windows and detached flake-like particles of Al-MCM-41-like phase.
4. ZSM5-MT(0.8): flake-like particles of Al-MCM-41-like phase.

**Figure 2:**
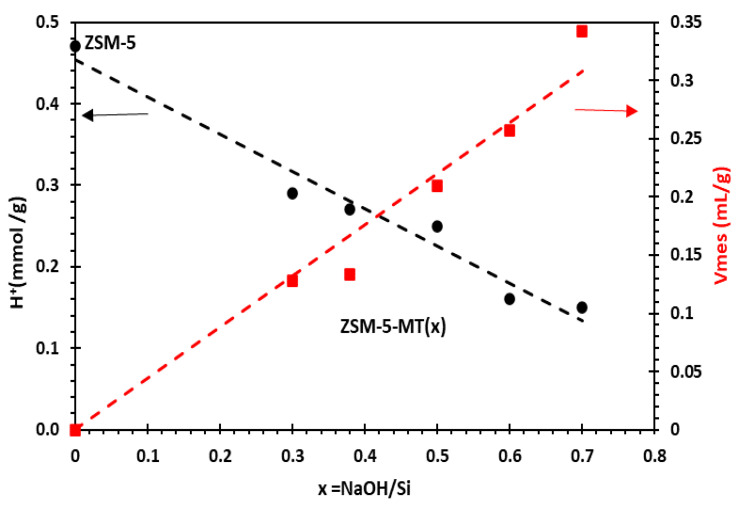
Evolution of mesoporous volume and number of Brønsted acid sites in mesoporous ZSM5-MT(x) as a function of x = NaOH/Si molar ratio in gel synthesis.

Nitrogen sorption isotherms at 77 K and a corrected t-plot method were used to precisely characterize the texture (micro- and mesopore volumes and surface areas) of ZSM5-MT(x) (Table S4) [18]. ZSM5-MT(x) are micro-/mesoporous catalysts with cylindrical mesopores with a 4.1 nm diameter and narrow pore size distribution as Al-MCM-41 materials [18]. Horizontal hysteresis on nitrogen isotherms of ZSM5-MT(x) is the signature of the cavities due to the hollow boxes that are connected to the exterior of the crystals by either micropores of ZSM-5- or Al-MCM-41-like mesopores, leading to a cavitation phenomenon (with a closure point at p/p_0_ = 0.47) (Figure S3). The highest width of the hysteresis for ZSM5-MT(0.5) is attributed to the thinner shells (thickness of 8 nm) of the hollow boxes in comparison to those of ZSM5-MT(0.38) (thickness of 20 nm), leading to a larger volume of cavities (Figure 1). While the micropore volume and surface area globally decrease with an increasing x (i.e., the NaOH/Si molar ratio in gel synthesis), the opposite trend was observed for the mesopore volume and surface area. For example, for ZSM5-MT(x) with x = 0.30, 0.38, and 0.50, the micropore volumes were 0.154, 0.135, and 0.122 mL g^−1^, respectively, and the mesopore volume (of the ordered mesopores of 4 nm) increased to 0.128, 0.133, and 0.209 mL g^−1^, respectively. ZSM5-MT(x), with x = 0.38, exhibited similar micropore and mesopore volumes. The mesopore volume of ZSM5-MT(x) increased linearly by increasing x (Figure 2), and so should the mass transfer of reactants and products [23].

The number of Brønsted acid sites was measured by FTIR/Pyridine after outgassing pyridine at 150 °C (Table S4) [19]. Although chemical analysis (estimated by ICP-OES) gave a global Si/Al ratio of 16 for all ZSM5-MT(x), the number of Brønsted acid sites decreased with x, whereas the mesoporous volume increased (Figure 2). ZSM5-MT(x), with x = 0.30, 0.38, and 0.50, featured a number of Brønsted acid sites of 0.29, 0.27, 0.25 mmol H^+^ g^−1^, respectively, whereas ZSM-5 exhibited 0.47 mmol H^+^ g^−1^ (Table S4).

The number of acid sites of all ZSM5-MT(x) remained, however, larger than a purely mesoporous amorphous Al-MCM-41 (Si/Al = 15), exhibiting only 0.05 mmol H^+^ g^−1^ [19]. The strength of the acid sites was evaluated by NH_3_-TPD (Figure S5, Table S4). The NH_3_-TPD spectra of ZSM5-MT(x) feature two peaks as the parent ZSM-5. The peak at a high temperature (T_2_) was relative to the strong acid strength of the catalyst. ZSM5-MT(0.3 and 0.38) featured the same strong acid strength as ZSM-5, with T_2_ = 420 °C. For the other ZSM5-MT(x) (x ≥ 0.5), the strength of the acid sites decreased with x.

### 3.3. Alkylation of Toluene by Methanol with ZSM-5 and Mesoporous ZSM-5 Catalysts at Low Contact Time (0.03 s)

**Figure 3:**
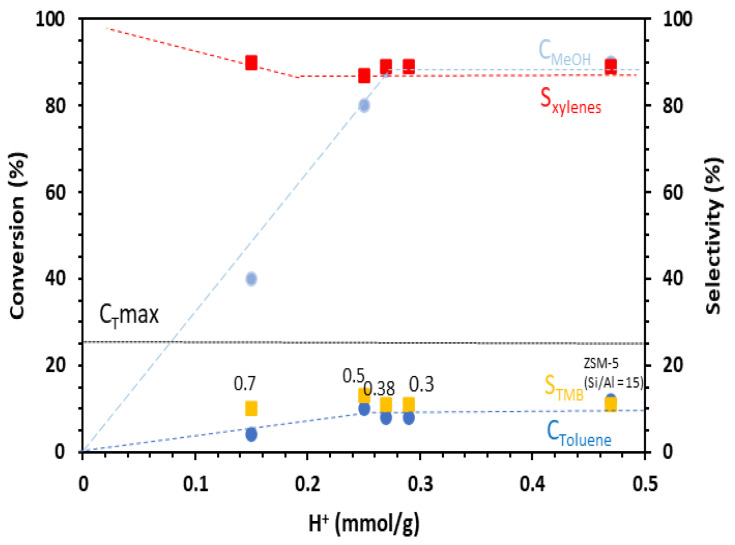
Conversion of toluene and methanol, selectivity in xylenes, and TMB as a function of the number of Brønsted acid sites of the catalysts in the alkylation of toluene by methanol. Conditions: toluene/methanol = 4/1, 10 mg catalyst + 140 mg quartz, contact time = 0.03 s, T = 350 °C, TOS = 15 min. Catalysts: commercial ZSM-5 (Si/Al = 15) and hierarchical ZSM5-MT(x). The value of x is indicated next to the data points.

The effect of mesoporosity in mesoporous ZSM-5 catalysts on toluene was examined in conditions favoring p-xylene formation, as presented previously (Section 3.1): T = 350 °C, contact time = 0.03 s, toluene/methanol = 4/1. We note that for ZSM-5 (Si/Al = 15) and all hierarchical ZSM5-MT(x) catalysts, the selectivity in xylenes was very high (90%), while the selectivity in TMB was only 10% (Figure 3, Table S5).

**Figure 4:**
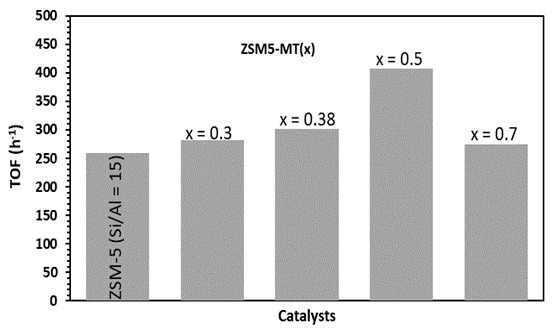
Turn over frequency (TOF) of toluene in the alkylation of toluene by methanol. Conditions: toluene/methanol = 4/1, 10 mg catalyst + 140 mg quartz, contact time = 0.03 s, T = 350 °C, TOS = 15 min. Catalysts: Commercial ZSM-5 Si/Al = 15 and hierarchical ZSM5-MT(x).

The data plotted in Figure 3 show that the toluene and methanol conversions increased with the number of Brønsted acid sites prior to reaching a plateau. The catalysts should feature a number of Brønsted acid sites higher than 0.25 mmol H^+^ g^−1^ (such as for ZSM5-MT(0.5)) to reach the highest toluene conversion observed, which was 10%, and to consume the maximum amount of methanol (90%). The toluene conversion decreased rapidly with the decrease in the Brønsted acid sites. ZSM5-MT(0.7), with 0.15 mmol H^+^ g^−1^, featured a toluene conversion of 4%. Purely mesoporous Al-MCM-41 (Si/Al = 15), with 0.05 mmol H^+^ g^−1^, reached only 1% toluene conversion even with a higher amount of catalyst (40 mg). Note that because of the toluene/methanol ratio of 4/1, the conversion of toluene cannot exceed 25%. In the conditions we chose to be out of thermodynamical equilibrium, mesoporous ZSM-5 did not increase the toluene conversion (10% for ZSM5-MT(0.5) vs. 12% for ZSM-5), xylene selectivity (87% for ZSM5-MT(0.5) vs. 89% for ZSM-5), or methanol alkylation efficiency (60% for ZSM5-MT(0.5) vs. 63% for ZSM-5) (Table S5) in comparison to the parent ZSM-5 nanocrystals, as it was noticed in the literature for catalytic reactions under thermodynamic equilibrium and a similar amount of Brønsted acid sites [17]. Figure 4 compares the performances of the catalysts expressed in terms of TOF (mole of toluene converted per mole of acid sites and time). We can observe that the TOF values exhibited by the ZSM5-MT(x) samples were higher than those exhibited by the parent ZSM-5. Moreover, the TOF increased as the x increased up to 0.5. This is because the mesopores created in ZSM5-MT(x) increased the accessibility of the reactants to the acid sites, and therefore, their conversion. The smaller TOF value observed for the ZSM5-MT(0.7) sample can be explained by the fact that this catalyst contains acid sites with lower strength than the other mesoporous ZSM-5 (Figure S5). Note that ZSM5-MT(0.5) was also the best catalyst (highest conversions) for the esterification of benzyl alcohol with hexanoic acid [19].

**Figure 5:**
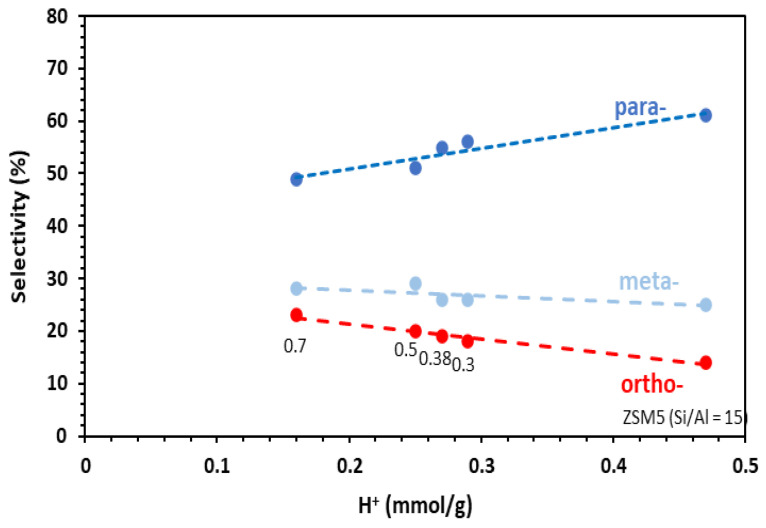
Selectivity in para-, meta-, ortho-xylenes as a function of the number of Brønsted acid sites of the catalysts in the alkylation of toluene by methanol. Conditions: toluene/methanol = 4/1, 10 mg catalyst + 140 mg quartz, contact time = 0.03 s, T = 350 °C, TOS = 15 min. Catalysts: commercial ZSM-5 Si/Al = 15 and hierarchical ZSM5-MT(x). The value of x is indicated under the data options.

The distribution of the xylene distribution is given in Figure 5. Accordingly, the para-selectivity can be related to the number of the Brønsted acid sites. At the same time, the mesoporous volume of the catalysts increased when the number of the Brønsted acid decreased (Figure 2). In fact, the mesopores created in ZSM5-MT(x) increase the accessibility of the reactants to the acid sites, and therefore, their conversion. On the other hand, the large pores promote the formation of o-xylene as a primary product (according to the stepwise mechanism [22]). Note that the toluene alkylation was carried out at a very low contact time (0.03 s). Under these conditions, the isomerization of the primary xylene isomers was avoided.

**Figure 6:**
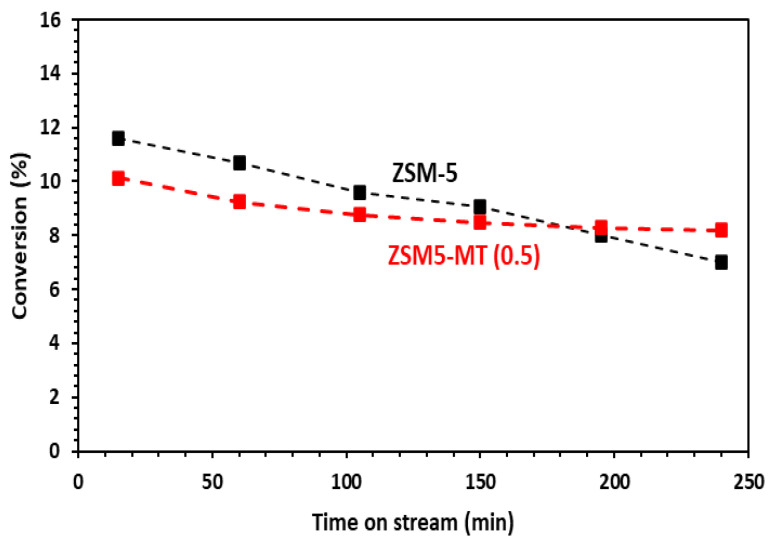
Conversion of toluene as a function of time on stream (TOS) in the alkylation of toluene by methanol. Conditions: toluene/methanol = 4/1, 10 mg catalyst + 140 mg quartz, T = 350 °C, TOS = 15 min. Catalysts: commercial ZSM-5 Si/Al = 15 and hierarchical ZSM5-MT(0.5). The results with the other ZSM5-MT(x) are given in Figure S3.

The above presented results were obtained after 15 min of reaction. For a longer reaction time, the ZSM-5 nanocrystals showed a continuous deactivation due to coke formation, with a loss of 5% toluene conversion after 4 h on stream, whereas all mesoporous ZSM5-MT(x) featured only 2% loss (Figure 6 and Figure S6). In terms of the relative loss in comparison to the starting toluene conversion, this represents losses of 39% for ZSM-5, 28% for ZMS5-MT(0.3), 19% for ZSM5-MT(0.5), and 45% for ZSM5-MT(0.7). The deactivation was lower in the case of mesoporous ZSM5-MT(0.5). Note also that the para-xylene selectivity did not change significantly within the time on stream for both ZSM-5 and ZSM5-MT(0.5) (Figure S7).

The mesopores bring a better diffusion of the products outside the acid sites and the zeolite channel, reducing the rate of coke formation, and slowing down the deactivation of the acid sites.

Based on these results, the mesoporous ZSM-5 does not allow for increasing the selectivity in p-xylene but should be a solution to extend the lifetime of the catalyst.

## 4. Conclusions

A series of mesoporous ZSM-5 catalysts prepared by micelle-templating from commercial ZSM-5 nanocrystals (Si/Al = 15) were tested in the alkylation of toluene by methanol. The main goal of this study was to examine the effect of mesopores on the para-xylene selectivity in toluene alkylation by methanol. No favorable effect of the mesopores on the para-selectivity was observed with mesoporous ZSM5-MT(x). However, mesoporous ZSM-5, especially ZSM5-MT(0.5), showed a better catalyst lifetime than the commercial ZSM-5. This behavior can be ascribed to a better diffusion of molecules, limiting the coke formation on the active sites. Mesoporous ZSM-5 should be associated with another strategy of ZSM-5 shape selectivity as external acid site passivation to promote para-selectivity and the catalyst lifetime at the same time.

## Data Availability

Not applicable.

## Supplementary Materials

The following supporting information can be downloaded at: https://www.mdpi.com/article/10.3390/ma16216872/s1.
