# Supplementary material for: Influence of the Mesoporosity of Hierarchical ZSM-5 in Toluene Alkylation by Methanol
Source: Materials (Basel). 2023 Oct 26;16(21):6872. doi: 10.3390/ma16216872 (PMC10649414; doi:10.3390/ma16216872)
Supplement: Supplementary file 1 [file materials-16-06872-s001.zip › materials-2682433-supplementary.pdf]

*Supplementary information*

# Influence of the Mesoporosity of Hierarchical ZSM-5 in Toluene Alkylation by Methanol

**Lucie Desmurs, Claudia Cammarano, Olinda Gimello, Anne Galarneau and Vasile Hulea ***

Charles Gerhardt Institute of Montpellier, University of Montpellier, CNRS, ENSCM, 1919 Rte de Mende, CEDEX 5, 34293 Montpellier, France; lucie.desmurs@enscm.fr (L.D.); claudia.cammarano@enscm.fr (C.C.); olinda.gimelo@enscm.fr (O.G.); anne.galarneau@enscm.fr (A.G.)

* Correspondence: vasile.hulea@enscm.fr

**Table S1.** Effect of temperature in the alkylation of toluene by methanol with ZSM-5 (Si/Al = 15).

| **T (°C)** | **Conversion (mol%)** | | **Aromatic selectivity (mol%)** | | **Xylene selectivity (%)** | | | $M_{alk}$ (%) | $R_{p-X}$ (%) | $R_{HC}$ |
|---|---|---|---|---|---|---|---|---|---|---|
| | $C_T$ | $C_M$ | X | TMB | p-X | m-X | o-X | | | |
| 350[a] | 76 | 100 | 51 | 49 | 24 | 52 | 24 | 26 | 9 | 1 (ref.) |
| 400[a] | 80 | 100 | 53 | 47 | 25 | 51 | 24 | 27 | 11 | 1.8 |
| 450[a] | 84 | 100 | 54 | 46 | 25 | 51 | 24 | 29 | 11 | 1.8 |
| 250[b] | 2 | 54 | 64 | 36 | 30 | 28 | 42 | 3 | 0 | 1 (ref.) |
| 300[b] | 14 | 61 | 61 | 39 | 31 | 27 | 41 | 17 | 3 | 1.6 |
| 350[b] | 38 | 60 | 59 | 41 | 35 | 39 | 26 | 47 | 8 | 3.1 |

[a] 200 mg ZSM-5, toluene/methanol = 1/4; [b] 80 mg ZSM-5, toluene/methanol = 1/2. Time on Stream (TOS) = 105 min. $C_T$: toluene conversion; $C_M$: methanol conversion; B: benzene; X: xylene; $M_{alk}$: methanol converted in alkylation compared to total converted metanol; Rp-X: yield in p-Xylene; $R_{HC}$: light hydrocarbons produced compared to 1, which was considered a reference value.

**Table S2.** Effect of molar ratio toluene/methanol in the alkylation of toluene by methanol with ZSM-5 (Si/Al =15).

| **T/M** | **Conversion** | | | | **Aromatic selectivity** | | **Xylene selectivity** | | | $\mathbf{M_{alk}}$ | $\mathbf{R_{p-X}}$ | $R_{HC}$ |
|---|---|---|---|---|---|---|---|---|---|---|---|---|
| | **(%)** | | | | **(%)** | | **(%)** | | | **(%)** | **(%)** | |
| | $C_T$ | $C_{T-max}$ | C | $C_M$ | X | TMB | p-X | m-X | o-X | (%) | (%) | |
| 0.5 | 38 | 100 | 38 | 63 | 59 | 41 | 35 | 39 | 26 | 44 | 7.8 | 18.3 |
| 2 | 27 | 50 | 54 | 92 | 74 | 26 | 38 | 44 | 18 | 75 | 7.6 | 3.3 |
| 4 | 17 | 25 | 68 | 100 | 82 | 18 | 27 | 52 | 21 | 87 | 3.8 | 1 (ref.) |

T = 350 °C; TOS = 105 min; 80 mg ZSM-5; 80 mL/min $N_2$. $C_T$: toluene conversion; $C_{T-max}$: maximum toluene conversion allowed by toluene/methanol ratio; C = $C_T$ / $C_{T-max}$; $C_M$: methanol conversion; B: benzene; X: xylene; $M_{alk}$: methanol converted in alkylation compared to total converted methanol; Rp-X: yield in p-Xylene; $R_{HC}$: light hydrocarbons produced compared to the T/M ratio of 4, considered as a reference.

**Table S3.** Effect of contact time in the alkylation of toluene by methanol with ZSM-5 (Si/Al =15).

| **Total gas flow (mL/min)** | **Catalyst mass (mg)** | **Contact time (s)** | **Conversion (%)** | | **Aromatic selectivity (% mol)** | | | Xylene selectivity (%) | | | $M_{alk}$ (%) | Rp-X (%) | $R_{HC}$ |
|---|---|---|---|---|---|---|---|---|---|---|---|---|---|
| | | | $C_T$ | $C_M$ | B | X | TMB | p-X | m-X | o-X | | | |
| 85 | 200 | 0.23 | 21 | 100 | 1 | 74 | 25 | 24 | 53 | 23 | 100 | 3.7 | 1 (ref.) |
| 85 | 80 | 0.09 | 17 | 100 | 0 | 82 | 18 | 27 | 52 | 21 | 87 | 3.8 | 0.8 |
| 85 | 10 +140 quartz | 0.03 | 12 | 90 | 0 | 89 | 11 | 61 | 25 | 14 | 63 | 5.5 | - |

TOS = 105 min; T = 350 °C; toluene/methanol = 4/1; $C_T$: toluene conversion ($C_{T-max}$ = 25%); $C_M$: methanol conversion; B: benzene; X: xylene; $M_{alk}$: methanol converted in alkylation compared to total converted methanol; $R_{p-X}$: p-Xylene yield; $R_{HC}$: light hydrocarbons produced compared to a reference. light hydrocarbons produced compared to the contact time of 0.23 s, considered as a reference.

**Table S4.** Textural and acidic properties of ZSM5-MT(x) catalysts. Comparison with parent ZSM-5 (Si/Al = 15).

| **Catalysts** | **$D_{mes}$ nm** | **$S_{BET}$ $m^2\,g^{-1}$** | **$S_{mic}$ $m^2\,g^{-1}$** | **$S_{mes+ext}$ $m^2\,g^{-1}$** | **$V_{mic}$ mL $g^{-1}$** | **$V_{mes}$ mL $g^{-1}$** | **TPD-$NH_3$ $T_1$ °C** | **$T_2$ °C** | **$Q_1$ mmol $g^{-1}$** | **$Q_2$ mmol $g^{-1}$** | **FTIR/pyridine Brønsted mmol $g^{-1}$** | **Lewis mmol $g^{-1}$** |
|---|---|---|---|---|---|---|---|---|---|---|---|---|
| ZSM-5 | - | 372 | 320 | 53 | 0.162 | 0 | 202 | 420 | 0.64 | 0.21 | 0.47 | 0.11 |
| ZSM5-MT(0.30) | 4.1 | 443 | 310 | 170 | 0.154 | 0.128 | 198 | 420 | 0.45 | 0.19 | 0.29 | 0.17 |
| ZSM5-MT(0.38) | 4.1 | 477 | 270 | 170 | 0.135 | 0.133 | 198 | 420 | 0.34 | 0.16 | 0.27 | 0.18 |
| ZSM5-MT(0.50) | 4.1 | 486 | 250 | 250 | 0.122 | 0.209 | 198 | 390 | 0.30 | 0.16 | 0.25 | 0.18 |
| ZSM5-MT(0.60) | 4.1 | 533 | 218 | 315 | 0.108 | 0.257 | 187 | 370 | 0.24 | 0.16 | 0.16 | 0.16 |
| ZSM5-MT(0.70) | 4.1 | 548 | 133 | 415 | 0.068 | 0.342 | 187 | 350 | 0.12 | 0.13 | 0.15 | 0.09 |

**Table S5.** Alkylation of toluene by methanol with ZSM-5 (Si/Al = 15) and ZSM5-MT(x) with 0.03 s contact time (10 mg catalyst + 140 mg quartz).

| **Catalysts** | **$H^+$ (µmol)** | **Conversion (%)** | | **Aromatic selectivity (mol%)** | | Xylene selectivity (%) | | | TOF ($h^{-1}$) | $M_{alk}$ (%) | $R_{p-X}$ (%) | $R_{HC}$ |
|---|---|---|---|---|---|---|---|---|---|---|---|---|
| | | $C_T$ | $C_M$ | X | TMB | p-X | m-X | o-X | | | | |
| ZSM-5 (Si/Al =15) | 4.7 | 12 | 90 | 89 | 11 | 61 | 25 | 14 | 259 | 63 | 6.5 | 1 (ref.) |
| ZSM5-MT(0.30) | 2.9 | 8 | 89 | 89 | 11 | 56 | 26 | 18 | 281 | 43 | 4 | 1.1 |
| ZSM5-MT(0.38) | 2.7 | 8 | 88 | 89 | 11 | 55 | 26 | 19 | 302 | 43 | 3.9 | 1 |
| ZSM5-MT(0.50) | 2.5 | 10 | 80 | 87 | 13 | 51 | 29 | 20 | 407 | 60 | 4.4 | 0.9 |
| ZSM5-MT(0.70) | 1.5 | 4 | 40 | 90 | 10 | 49 | 28 | 23 | 274 | 47 | 1.8 | 0.5 |

TOS = 15 min; $H^+$: number of Brønsted acid sites in the column; T = 350 °C; toluene/methanol = 4/1; $C_T$: toluene conversion ($C_{T-max}$ = 25%); $C_M$: methanol conversion; B: benzene; X: xylene; TOF: Turn Over Frequency of toluene; $M_{alk}$: methanol converted in alkylation compared to total converted methanol; $R_{p-X}$: p-Xylene yield; $R_{HC}$: light hydrocarbons produced compared to ZSM-5 (Si/Al = 15), considered as a reference.

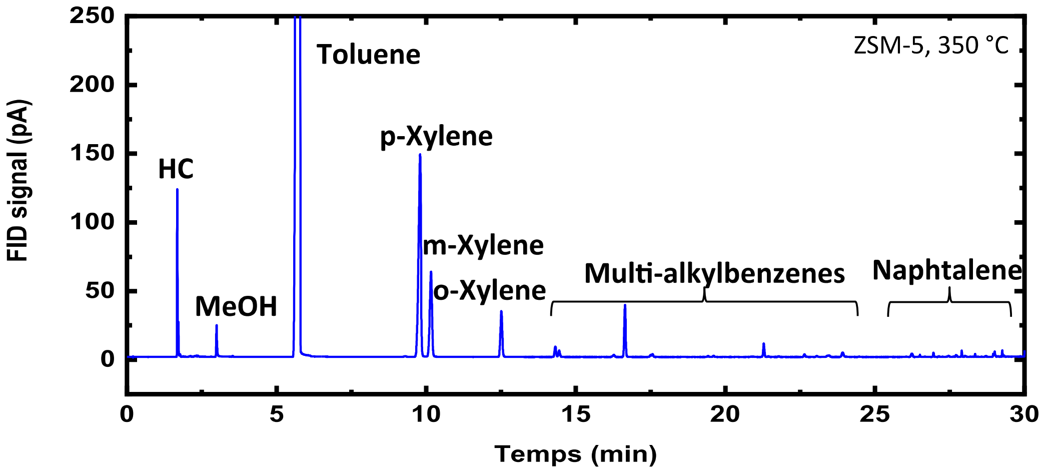


**Figure S1.** Typical chromatogram obtained in the alkylation of toluene by methanol (MeOH). Conditions: toluene/methanol = 4/1, 10 mg ZSM-5 (Si/Al = 15), T = 350 °C, contact time = 0.03 s, TOS = 15 min.

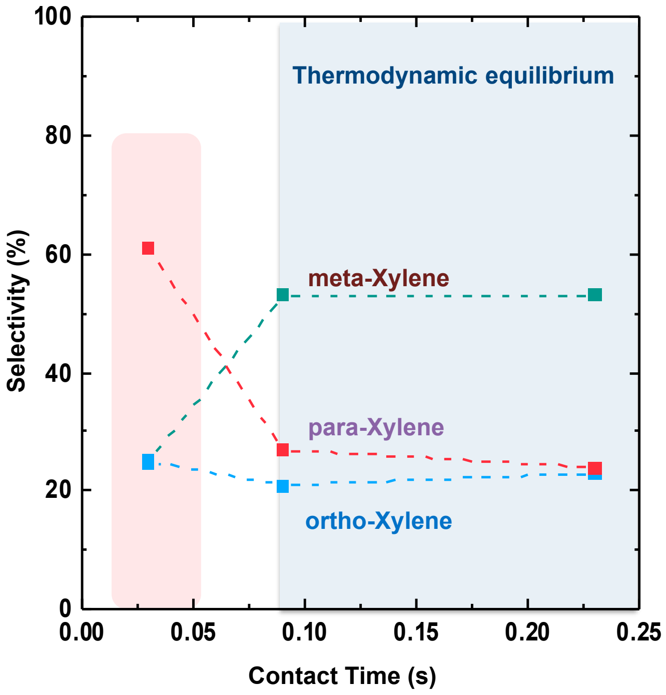


**Figure S2.** Selectivity in para-, meta-, ortho-xylenes in the alkylation of toluene by methanol as a function of contact time. Conditions: toluene/methanol = 4/1, ZSM-5 (Si/Al = 15), T = 350 °C, TOS = 105 min.

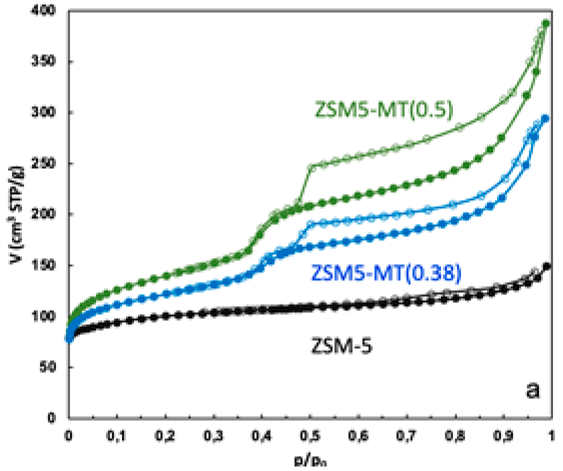


**Figure S3.** Nitrogen sorption isotherms of selected ZSM5-MT(x).

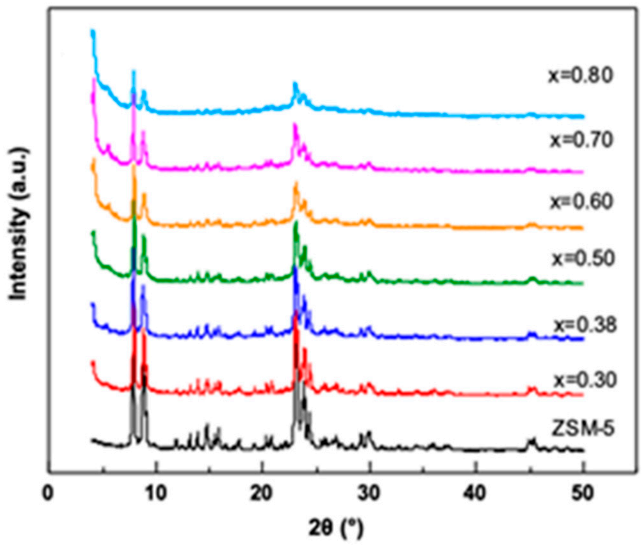


**Figure S4.** XRD pattern of ZSM5-MT(x) as a function of x = NaOH/Si molar ratio in the gel synthesis. Comparison with parent ZSM-5.

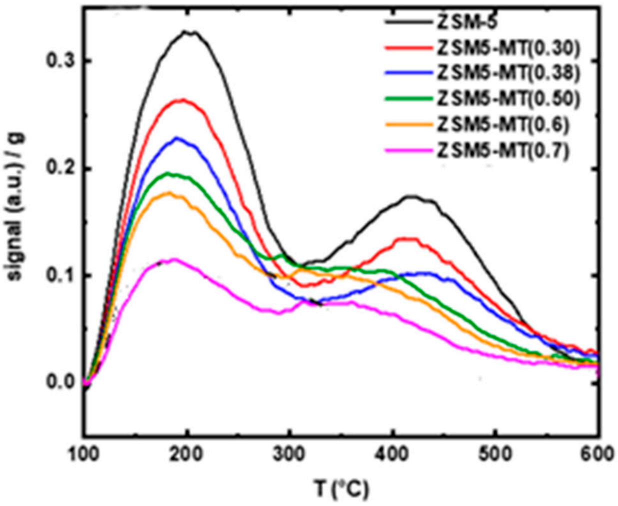


**Figure S5.** $NH_3$-TPD of ZSM5-MT(x) as a function of x = NaOH/Si molar ratio in the gel synthesis. Comparison with parent ZSM-5.

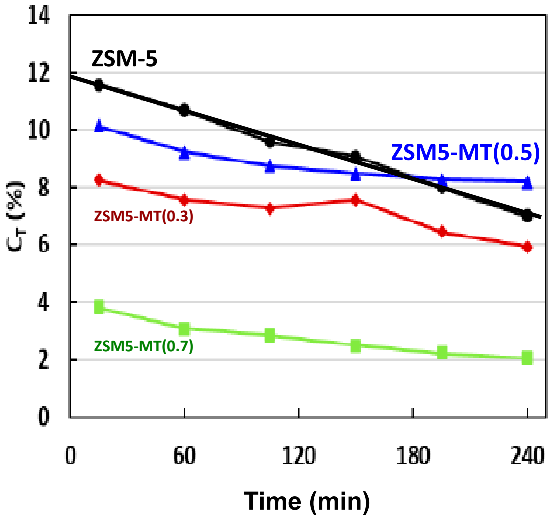


**Figure S6.** Toluene conversion as a function of time on stream (TOS) in the alkylation of toluene by methanol with ZSM-5 (Si/Al = 15) and ZSM5-MT(x). Conditions: toluene/methanol = 4/1, 10 mg catalyst + 140 mg quartz, T = 350 °C.

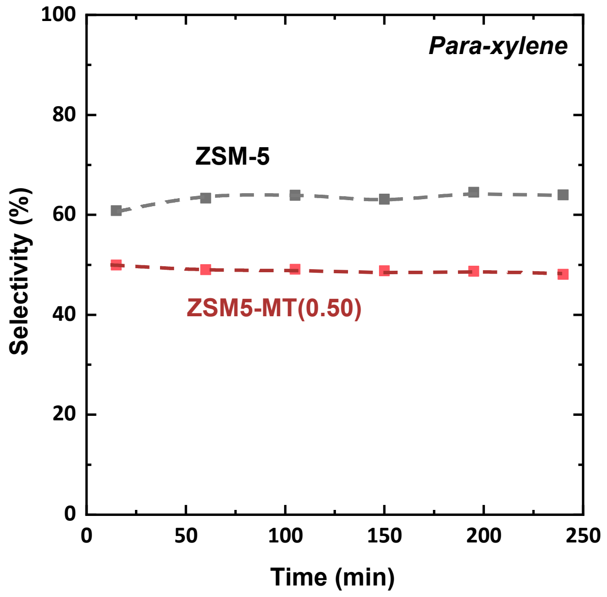


**Figure S7.** Evolution of the selectivity in para-xylene as a function of time on stream (TOS) in the alkylation of toluene by methanol with ZSM-5 (Si/Al = 15) and ZSM5-MT(0.5). Conditions: toluene/methanol = 4/1, 10 mg catalyst + 140 mg quartz, T = 350 °C.
